# Influence of Technological Parameters on Chip Formation and Chip Control in Precision Hard Turning of Ti-6Al-4V

**DOI:** 10.3390/mi14101973

**Published:** 2023-10-23

**Authors:** Elshaimaa Abdelnasser, Samar El-Sanabary, Ahmed Nassef, Azza Barakat, Ahmed Elkaseer

**Affiliations:** 1 Department of Production Engineering and Mechanical Design, Faculty of Engineering, Port Said University, Port Fouad 42526, Egypt; alshymaa.gamal@eng.psu.edu.eg (E.A.); samar.abaas@eng.psu.edu.eg (S.E.-S.); nassef12@eng.psu.edu.eg (A.N.); 2 Higher Institute for Electronic Engineering—K 10 Bilbies, 10th of Ramadan City 44629, Egypt; 3 Mechanical Engineering Department, Faculty of Engineering, Helwan, Helwan University, Cairo 11792, Egypt; barakatazza@h-eng.helwan.edu.eg; 4 Department of Mechanical Engineering, Faculty of Engineering, The British University in Egypt (BUE), El-Sherouk City 11837, Egypt; 5 Institute for Automation and Applied Informatics, Karlsruhe Institute of Technology, 76344 Karlsruhe, Germany

**Keywords:** precision hard turning, Ti-6Al-4V, chip evacuation control, chip morphology, process parameters, surface roughness

## Abstract

This article presents the results of an experimental investigation into the effect of process parameters in the precision hard turning of Ti-6Al-4V on chip morphology at both macro and micro levels. It also reports on the control of chip generation to improve chip evacuation and breakability at the macro level by varying the process parameters, namely, feed rate, cutting speed and depth of cut during turning tests. A scanning electron microscope (SEM) was used to examine the chips produced for a better understanding of chip curling mechanisms at the micro level. Surface roughness of the machined specimens was measured to assess the effect of chip evacuation on obtainable surface quality. From the results, it was found that the interaction of process parameters has a significant effect on the control of chip formation. In particular, the interaction of higher cutting speeds and greater depths of cut produced chip entanglement with the workpiece for all values of feed rates. Using relatively higher feed rates with a low depth of cut showed good results for chip breaking when machining at higher cutting speeds. Different chip curling mechanisms were identified from the SEM results. Chip side-curl formation showed different segmentation patterns with an approximately uniform chip thickness along the chip width, while chip up-curl occurred due to variations in chip thickness. Finally, it was found that the tangling of the chip with the workpiece has a significant effect on the final surface quality.

## 1. Introduction

When assessing machine attributes in regard to metal cutting, chip morphology is one of the most important factors [1]. Chip formation reflects the underlying mechanisms of the cutting process and workpiece–tool interaction in the cutting zone [2] and is affected by machining parameters such as feed, cutting speed and depth of cut [3], as well as thermal behavior during machining and friction between tool rake face and deformed chips [4]. Chip formation is directly related to surface quality, tool wear and machining efficiency [5,6]. Thus, the control of the chip formation mechanism at both micro and macro levels is a possible means to improve machinability [7].

Continuous unbroken chips have been found to be problematic when machining ductile materials such as titanium-based alloys, especially in turning operations [8]. In fact, it is the generation of these problematic continuous chips that causes titanium alloys to be classified as difficult to cut materials [9]. Long unbroken chips are considered a major obstacle to automated machining systems as the chips can wrap around and become entangled with the workpiece or cutting tool, even becoming squeezed between them and causing deterioration of the final machined surface [10]. Such events make it necessary to stop machining, which hinders the machining process, decreasing productivity and surface quality and increasing operational costs [11]. Additional adverse factors include harm to machine operators and damage to the workpiece, cutting tool and machine tool, resulting in a shorter tool life [12,13]. Such considerations have promoted research into chip control to overcome the problems caused by continuous long chips [14].

**Figure 1:**
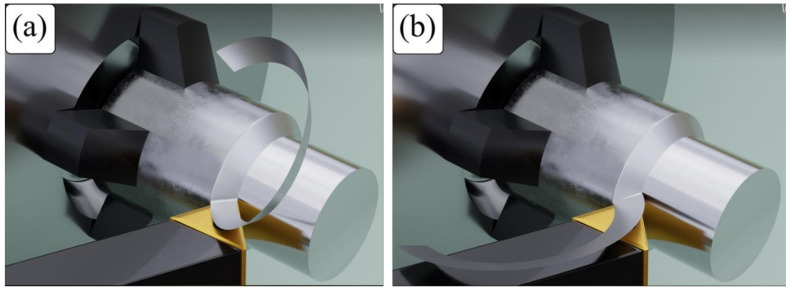
Two basic modes of chip curling; (**a**) chip up-curl and (**b**) chip side-curl.

Chip control means the control of chip curling, chip evacuation and chip breaking [15]. Chips start to curl as they leave the rake face of the tool and has two basic modes [16], as shown in Figure 1: chip up-curl and chip side-curl. According to Fang [17], an up-curl chip is formed when the chip curls in the direction of chip thickness, around a horizontal central line (see Figure 1a). This shape of chip forms due to the difference in metal flow velocity between the top (free) surface and bottom surface of the chip, which is in contact with the tool rake face. A side-curl chip is formed when the chip curls in the direction of chip width, around a vertical central line, as illustrated in Figure 1b, and is produced by the variation of chip flow velocity along the direction of the chip width.

At the macro level, chip formation and flow is due the given cutting conditions and subject to chip curling [18], chip flow angle [19] and chip curve radius [2]. As a result, various shapes of chip can be formed, such as helical, tubular, ribbon, spiral, segmented and snarled [12,13,20].

The effect of cutting conditions on chip curling has received much attention, and it has been found that increasing the feed rate and depth of cut increased chip curling [14]. Fang [18] studied chip control through a theoretical investigation and reported that a higher cutting speed and lower feed rate may prevent chip curling, and increasing chip thickness increased chip curling. A number of studies have been conducted on chip flow angle, and it has been reported that tool geometry, feed rate and depth of cut have a great influence on chip flow angle [21,22]. Devotta et al. [2] studied chip curvature, produced experimentally and by finite element simulation on steel alloys, and found that chip curvature increased with an increase in feed rate and rake angle.

Chip breaking during the cutting process can be classified into two types: natural and forced. The first type occurs when the chip self-breaks without touching any obstacle, while the second one takes places when the chip is in contact with the tool or workpiece [13]. Research has indicated that chip curve radius is one of the most significant parameters that affects chip breaking, as decreasing the chip radius encourages the chip to break due to the bending moment involved [23]. Bending of the chip results in strains due to compression of the free surface and tension in the back surface, which is responsible for forming chip up-curl. Chip breaking occurs due to the tension stress on the outer surface of the curved chip. A smaller radius of chip curvature increases the difference between the tension and compression stresses, resulting in increased strains and leading to fracture [23]. Nakayama [24] presented a model to predict the fracture strain based on chip thickness and chip curve radius and indicated that when the chip flow on the tool rake face had an up-curl radius, it is continuous until blocked by the workpiece. Chip breaking occurs due to a bending moment in the opposite direction as a result of the reaction of the workpiece on the chip. Li [25] concluded that the chip will always break when the feed rate and depth of cut is higher than certain critical values; otherwise, the chip will not break. Fang [18] studied chip twisting due to high bending and torsional deformations and reported that chip twisting encouraged chip breaking and was affected by the radius of chip curvature and the pitch of the helix of a helical chip (the distance between two rings).

Several methods have been used to control chip flow and chip breaking for more efficient machining processes. Some studies investigated the effect of machining parameters on chip control, such as Zhang et al. [26] who reported that low cutting speed resulted in continuous chips when machining AISI 1045 steel and high-speed machining encouraged chips to break due to the formation of a serrated chip. Yilmaz et al. [14] reported that chip breaking takes place more easily when increasing cutting speed, feed rates and depths of cut. It has also been found that cutting tool geometry and workpiece materials have a significant effect on chip control [27]. However, the conditions that gave better chip control tended to have a negative effect on surface roughness as a result of machining with a large feed rate and depth of cut, for example [14]. Others have used chip breaker inserts [11] or custom breaker apparatus [6] to control chip curling and breaking.

Various forms of chip breakers have been developed such as obstruction-type devices attached to the cutting insert [28] or groove-type chip breakers with varying geometrical shapes machined on the rake face where the tool and chip interface [29]. Kim et al. [30] developed a chip breaker performance tester, for which commercial cutting tools with different forms and shapes were utilized as input to train a neural network under various cutting conditions. The authors concluded that the performance of chip breakers significantly depends on process parameters, namely, feed rate and depth of cut, and applying the neural network method was found to be a reliable technique to test and evaluate the performance of different chip breakers. For the tailoring of chip breakers based on the machining parameters and work material properties, a deep understanding of chip flow and chip breaking behavior is required [31]. Although chip breakers offer a good solution for chip control, especially in finishing machining, they lead to increased tooling cost and difficulties in chip control when machining ductile materials such as titanium alloys [14].

As reported by Nath et al. [32], applying a coolant is one of the most important methods of chip control and provides good results for chip evacuation. When machining titanium alloys, a flood and cryogenic cooling system helped to convert snarled chips to helical chips, which is preferable, but tended not to break the chip, as titanium alloys are both high strength and ductile [8]. However, a high-pressure coolant was found to be effective for chip breaking when machining titanium alloys [33] but could cause notch wear and adversely affect tool life [14]. Other methods have been used during machining, such as vibration-assisted cutting [34], which proved a successful method of chip control but might have a negative effect on surface quality [14].

It is worth emphasizing that excessive tool wear rates during the machining of titanium alloys contribute to the challenges associated with working on these materials. Consequently, the careful selection of an appropriate cutting tool is essential to ensure both reliability and high productivity in machining processes. Ideal cutting tools for machining difficult-to-cut materials such as titanium alloys should possess characteristics such as the ability to maintain hardness at elevated temperatures, high thermal conductivity and exceptional wear resistance [35,36].

Researchers have extensively explored various cutting tool materials and coatings for machining titanium-based alloys. Among these investigations, it has been consistently demonstrated that polycrystalline diamond (PCD) tools exhibit outstanding performance when machining titanium. This superiority can be attributed to their remarkable thermal conductivity [8], which facilitates efficient heat dissipation from the cutting zone during machining. Diamond, being the hardest material available in the machining industry, equips PCD tools with exceptional wear resistance compared to other tool materials [37]. In practical applications, PCD tools consistently outperform other cutting tool materials, offering extended tool life and the capability to operate under higher cutting parameter values [8].

Hourmand et al. [38] extensively explored the impact of machining parameters on various aspects of titanium alloy during turning and milling. They found that segmented chip formation during turning generates cyclic forces, with the frequency and amplitude of these forces depending on the machining parameters.

Li et al. [39] utilized Abaqus finite element software to create a simulation model and investigated serrated chip formation at different tool rake angles and cutting speeds, offering theoretical insights for Ti-6Al-4V cutting. Additionally, they studied stress and strain characteristics within adiabatic shear bands, concluding that serrated chip formation in titanium alloy machining leads to significant changes in shear strain and stress within adiabatic shear bands, particularly when rake angles range from 0° to 5°.

López de Lacalle et al. [40] compared a conventional flood coolant with minimal quantity lubrication (MQL) and liquid carbon dioxide (LCO2) during Ti-6Al-4V machining. Meanwhile, they explored the application of self-lubricating TiSiVN coatings on Al_2_O_3_–SiC ceramic cutting tools in dry cutting environments, considering both machining performance and Life Cycle Assessment (LCA) analysis [41]. Additionally, they investigated the effect of cutting tools on dry machining of Ti-6Al-4V, with and without TiSiVN coatings [42].

Wang et al. [43] studied serrated chip formation mechanisms in Ti-6Al-4V alloy under dry and liquid nitrogen cooling conditions, revealing that higher cutting speeds or lower temperatures increase chip serration and promote chip fracture.

Palanikumar et al. [44] investigated the impact of cutting conditions on cutting zone temperature when machining Ti-6Al-4V, using PVD carbide and CVD-coated tools. Their results demonstrated that cutting temperature increases with cutting speed, and CVD-coated tools produce shorter chips compared to PVD-coated tools.

Lv et al. [45] explored the influence of the cutting step on grain refinement and work hardening in machined Ti-6Al-4V alloy chips. They validated a two-step cutting finite element simulation model, revealing that higher cutting speeds result in smaller deformation zones, finer grain sizes and higher micro-hardness in the primary shear zone.

Denkena et al. [46] investigated chip formation in Ti-6Al-4V under different atmospheres during orthogonal cutting. They found that the surrounding atmosphere significantly affects chip formation, with non-periodic segmentation under oxygen-free conditions and segmental chip formation under air. The inert gas atmosphere reduced the feed force by up to 16.5% due to reduced friction.

Niu et al. [47] studied the impact of material constitutive laws on Ti-6Al-4V saw-tooth chip formation modeling. They proposed a joint material constitutive law (JC-TANH) combining advantages of the Johnson–Cook and TANH constitutive laws. Their research emphasized the importance of selecting the right constitutive law for accurate cutting simulation, with the JC-TANH model offering the best agreement with experimental results. Enhanced SPH methods were used to implement this joint constitutive model.

A literature review showed a gap in the investigation of chip control of titanium-based alloys and difficulty in evacuating break chips. There is not yet an adequate study on the effect of process parameters on chip control when machining titanium alloys. There remains a need for means by which to improve the machinability of Ti-6Al-4V alloy without adding to the cost, as incurred by using breakers or complex cooling systems. This research study aims to experimentally investigate chip control in turning Ti-6Al-4V by varying the process parameters to find their optimum ranges for chip evacuation and breaking and their effect on surface quality under dry machining conditions. In particular, the effect of the feed rate, cutting speed and depth of cut on chip morphology are studied at the macro level. The study is also enriched with an examination of chip morphology at the micro level by a SEM to achieve a better understanding of the relationship between chip formation and chip curling mechanisms. Surface roughness was used as an important measure of the effect of chip evacuation on the quality of the machined surface.

## 2. Experimental Work

Rods of Ti-6Al-4V alloy of 25 mm diameter and 150 mm length were used in the experimental tests. The measured hardness of specimens was (367 V_30_); the chemical composition of the Ti-6Al-4V alloy is presented in Table 1.

Turning operations were carried out on a CNC lathe (5.5 kW spindle motor, 6000 rpm maximum spindle speed). Polycrystalline diamond (PCD) inserts were used with a 0° rake angle, 11° clearance angle and 1.2 mm nose radius, for which a tool holder type (PDJNR 2020 K15) by Taegu Tec (Daegu, Republic of Korea) was used. All experiments were conducted under dry cutting conditions.

A full factorial design of the experiment was followed when carrying out the trials to investigate the effect of cutting speed, feed rate and depth of cut on chip morphology and its effect on the machined surface. The selection of parameter ranges was performed based on preliminary experiments aimed at evaluating chip control and surface roughness. As previously stated, this decision was extracted from the inherent challenges associated with chip evacuation during titanium turning, where the uninterrupted chip formation could become entangled around the workpiece, potentially compromising result accuracy. Table 2 presents all constant and variable machining conditions.

Photos of each experiment were taken by a high-quality digital camera to examine chip flow during machining. Scanning electron microscope (SEM) [SM 6380 LV, by Jeol, Frankfurt, Germany] was utilized for detailed studies of the chip morphology. A Surtronic (3+ stylus) profilometer was used to characterize the surface roughness of specimens; the cut-off length used was 0.8 mm. Five measurements of surface roughness (Ra) were taken under the same cutting condition and the arithmetic average was calculated. The arithmetic roughness average (Ra) was chosen to evaluate surface roughness, as it is the most commonly used surface roughness parameter in the industry for assessing surface quality.

## 3. Results and Discussion

**Figure 2:**
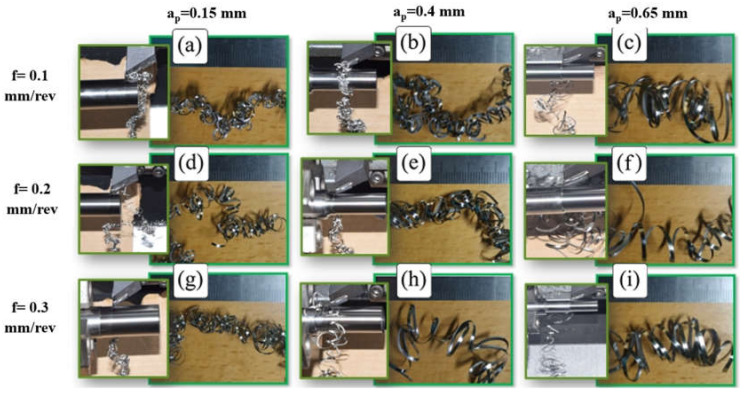
(**a**–**i**) Chip morphology during and after machining at the lowest v_c_ (75 m/min) with (**a**) 0.1 mm/rev f and 0.15 mm a_p_, (**b**) 0.1 mm/rev f and 0.4 mm a_p_, (**c**) 0.1 mm/rev f and 0.65 mm a_p_, (**d**) 0.2 mm/rev f and 0.15 mm a_p_, (**e**) 0.2 mm/rev f and 0.4 mm a_p_, (**f**) 0.2 mm/rev f and 0.65 mm a_p_, (**g**) 0.3 mm/rev f and 0.15 mm a_p_, (**h**) 0.3 mm/rev f and 0.4 mm a_p_, and (**i**) 0.3 mm/rev f and 0.65 mm a_p_.

**Figure 3:**
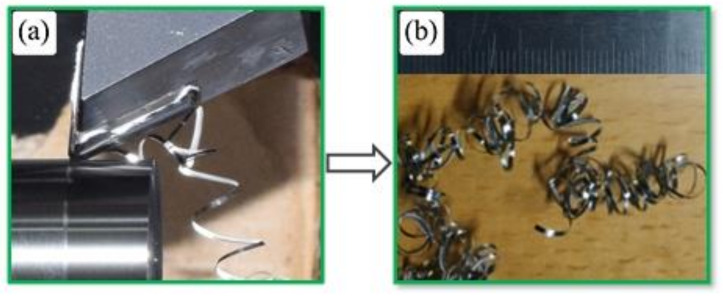
(**a**) Chip-up curl formation during machining and (**b**) chip produced at cutting speed 75 m/min, feed rate 0.2 mm/rev and depth of cut 0.15 mm.

**Figure 4:**
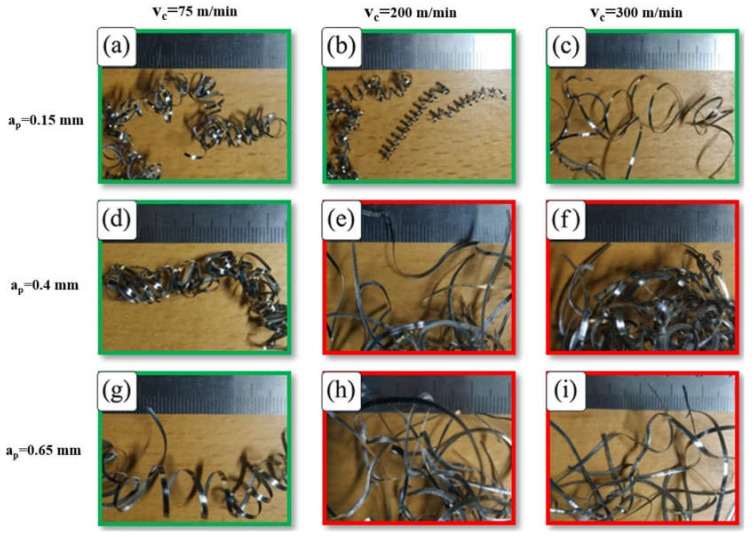
Chip morphology produced by turning tests at f of 0.2 mm/rev and (**a**) v_c_ of 75 m/min and 0.15 mm a_p_, (**b**) v_c_ of 200 m/min and 0.15 mm a_p_, (**c**) v_c_ of 300 m/min and 0.15 mm a_p_, (**d**) v_c_ of 75 m/min and 0.4 mm a_p_, (**e**) v_c_ of 200 m/min and 0.4 mm a_p_, (**f**) v_c_ of 300 m/min and 0.4 mm a_p_, (**g**) v_c_ of 75 m/min and 0.65 mm a_p_, (**h**) v_c_ of 200 m/min and 0.65 mm a_p_, and (**i**) v_c_ of 300 m/min and 0.65 mm a_p._

**Figure 5:**
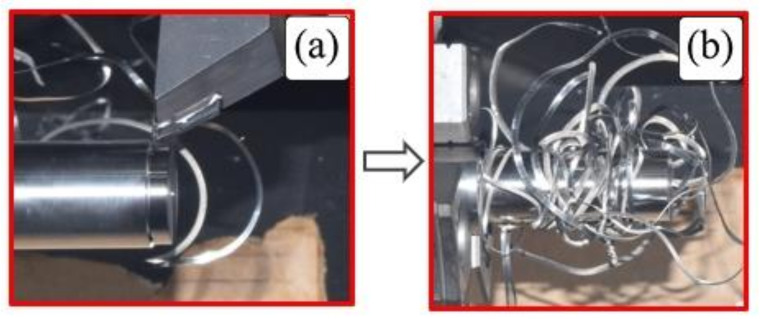
(**a**) Chip-side curl formation during machining and (**b**) consequent chip tangling with workpiece, at f 0.2 mm/rev, v_c_ 200 m/min and a_p_ 0.65 mm.

**Figure 6:**
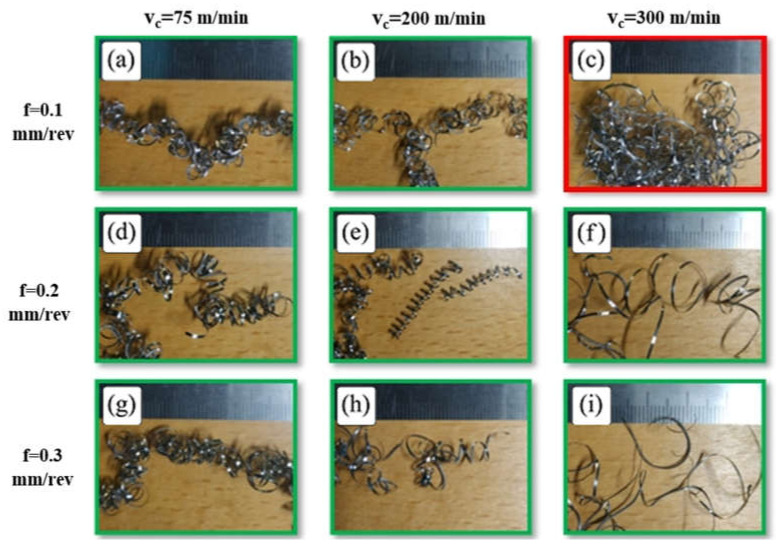
The effect of low depth of cut (0.15 mm) on chip morphology with (**a**) v_c_ of 75 m/min and 0.1 mm f, (**b**) v_c_ of 200 m/min and 0.1 mm f, (**c**) v_c_ of 300 m/min and 0.1 mm f, (**d**) v_c_ of 75 m/min and 0.2 mm f, (**e**) v_c_ of 200 m/min and 0.2 mm f, (**f**) v_c_ of 300 m/min and 0.2 mm f, (**g**) v_c_ of 75 m/min and 0.3 mm f, (**h**) v_c_ of 200 m/min and 0.3 mm f, (**i**) v_c_ of 300 m/min and 0.3 mm f.

**Figure 7:**
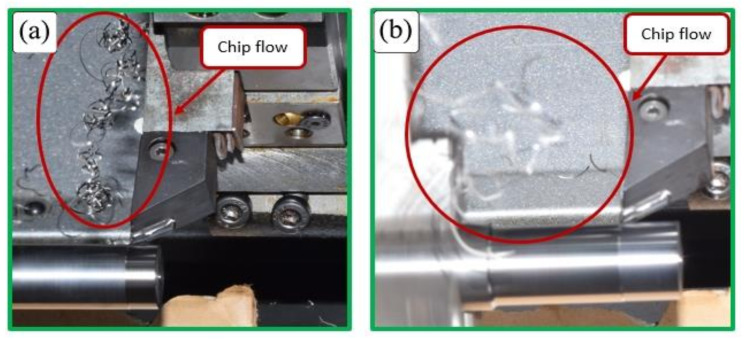
Chip breakage at low depth of cut (0.15 mm); (**a**) cutting speed = 200 m/min and feed rate = 0.3 mm/rev, and (**b**) cutting speed = 300 m/min and feed rate = 0.2 mm/rev.

**Figure 8:**
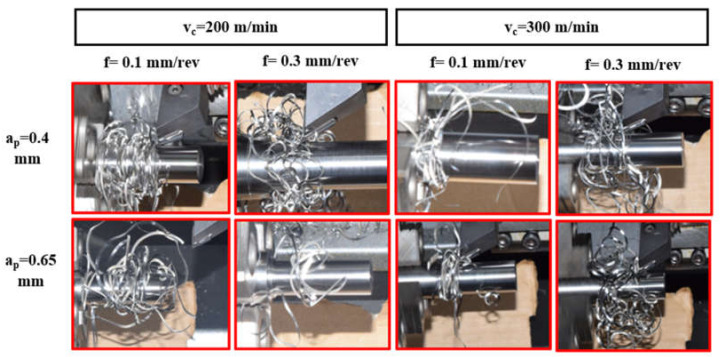
The effect of a combination of higher cutting speeds (200, 300 m/min) and higher depths of cut (0.4, 0.65 mm) at two feed rates (0.1, 0.3 mm/rev).

**Figure 9:**
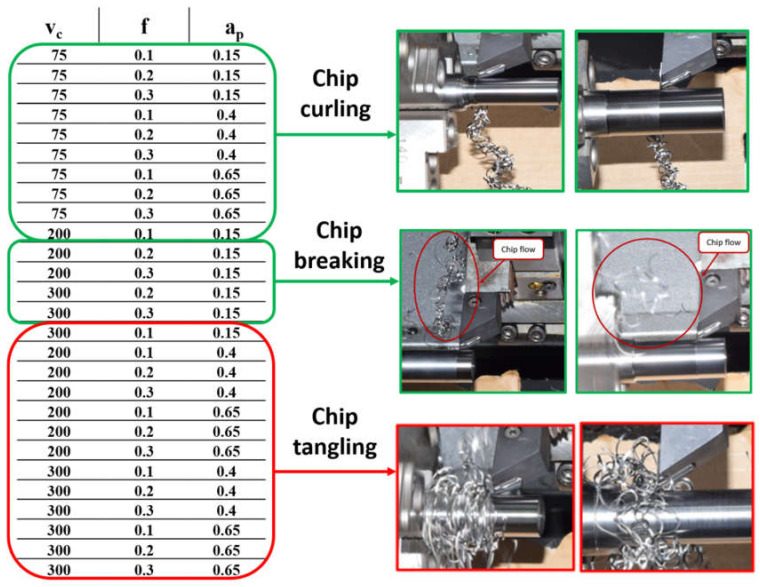
The safe (green) and risky (red) combinations of cutting speed, feed rate and depth of cut for chip evacuation.

**Figure 10:**
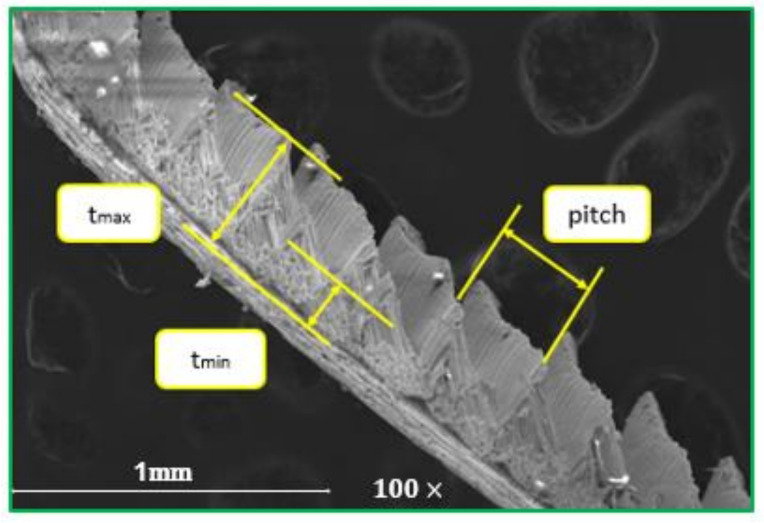
SEM image showing saw-tooth chip produced during machining Ti-6Al-4V alloys with cutting conditions (f = 0.2 mm/rev, v_c_ = 75 m/min and a_p_ = 0.65 mm).

**Figure 11:**
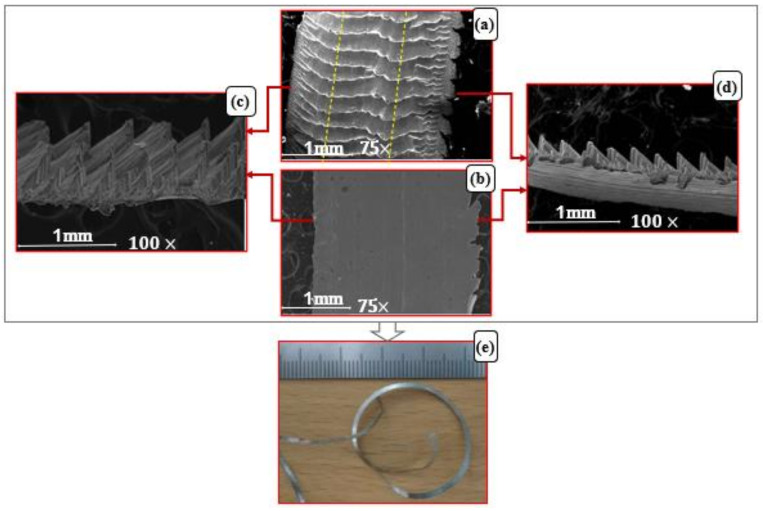
SEM image of chip morphology for side-curling chip produced at cutting conditions f = 0.3 mm/rev, v_c_ = 300 m/min and a_p_ = 0.65 mm; (**a**) free surface of chip at micro scale, (**b**) back surface of chip at micro scale, (**c**,**d**) two sides of chip thicknesses at micro scale and (**e**) chip shape at macro scale.

**Figure 12:**
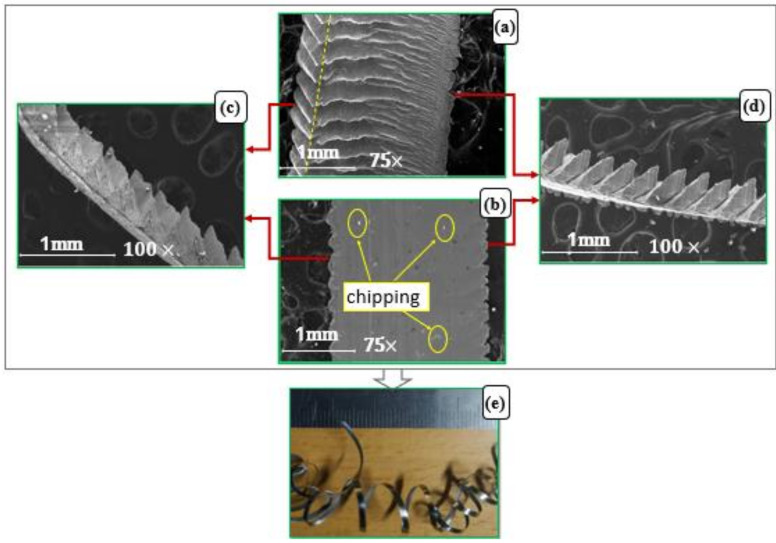
SEM image of chip morphology for an up-curling chip produced at cutting conditions f = 0.2 mm/rev, v_c_ = 75 m/min and a_p_ = 0.65 mm: (**a**) free surface of chip at micro scale, (**b**) back surface of chip at micro scale, (**c**,**d**) two sides of chip thicknesses at micro scale and (**e**) chip shape at macro scale.

**Figure 13:**
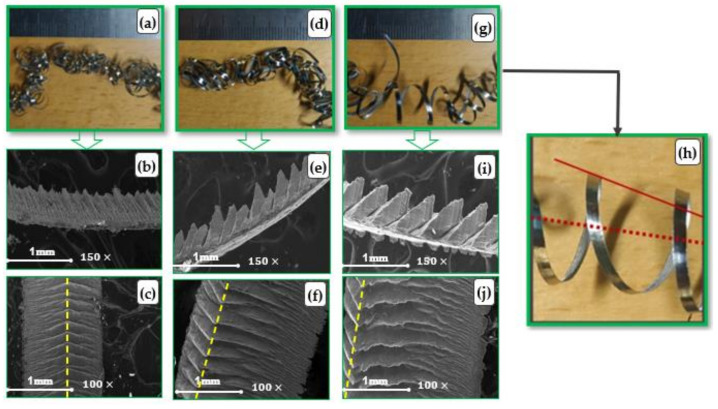
SEM images of chip morphology with helical shapes; at cutting conditions (f = 0.3 mm/rev, v_c_ = 75 m/min and a_p_ = 0.15 mm), where (**a**) chip shape at macro level, (**b**) chip side view at micro level and (**c**) chip free surface at micro level; at cutting conditions (f = 0.2 mm/rev, v_c_ = 75 m/min and a_p_ = 0.4 mm), where (**d**) chip shape at macro level, (**e**) chip side view at micro level and (**f**) chip free surface at micro level; and at cutting conditions (f = 0.2 mm/rev, v_c_ = 75 m/min and a_p_ = 0.65 mm), where (**g**) chip shape at macro level with zoom-in view presented in (**h**,**i**) chip side view at micro level and (**j**) chip free surface at micro level.

**Figure 14:**
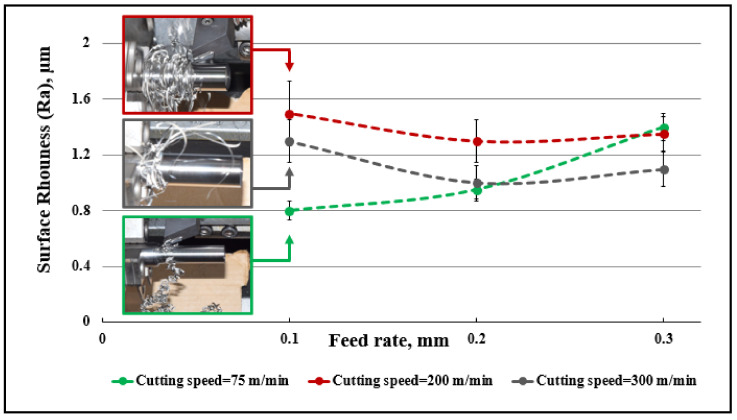
Effect of chip evacuation on measured surface roughness at different feed rates and cutting speeds with depth of cut 0.4 mm.

**Figure 15:**
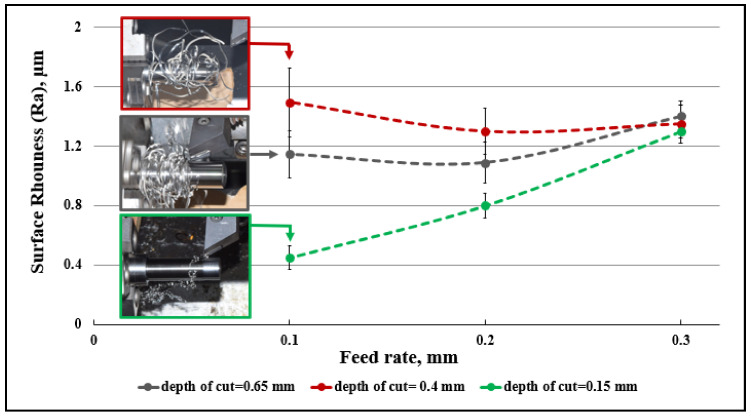
Effect of chip evacuation on measured surface roughness under different feed rates and depth of cut and v_c_ = 200 m/min.

This section discusses the experimental results in three sub-sections. First, the effect of process parameters on chip control at the macro level is presented. This is followed by a detailed comparison of chip morphology at micro and macro levels, and finally, the effect of chip control on generated surface roughness is given. The experimental results examined in this section are presented with a number of illustrations, Figure 2, Figure 3, Figure 4, Figure 5, Figure 6, Figure 7, Figure 8, Figure 9, Figure 10, Figure 11, Figure 12, Figure 13, Figure 14 and Figure 15, in which the performance of well-controlled chips under safe combinations of cutting parameters are highlighted in green, while uncontrolled chips due to risky combinations of process parameters are highlighted in red.

### 3.1. Effect of Process Parameters on Chip Control

Figure 2a–i shows chip morphology during and after turning Ti-6Al-4V alloy at a low cutting speed (v_c_ = 75 m/min) for the whole range of feed rates and depths of cut. It was found that machining at a low cutting speed produced continuous curled chips which flowed down without tangling with the tool or workpiece; thus, it achieved good chip evacuation. It was observed that chip up-curl was dominant at v_c_ = 75 m/min, as shown in Figure 3a, an enlarged image of the turning test when machining with feed rate = 0.2 mm/rev and depth of cut = 0.4 mm, which shows the beginning of the formation of chip up-curl during machining and which produced a helical chip, Figure 3b.

To better understand chip evacuation in this case (v_c_ = 75 m/min), the chip shape was examined for all feed rates and depths of cut, as illustrated in Figure 2. It was found that chip curling increased with an increased feed rate and depth of cut, with the interaction of higher values of feed rate and depth of cut leading to helical chips, which agree with the findings reported in [14]. Looking at Figure 2a, with the lowest feed rate (0.1 mm/rev) and depth of cut (0.15 mm), it is not difficult to see that the chip curling is a combination of up-curl and side-curl, with the side-curl dominant and with a small chip curvature. As a result, the chip has tangled rings which prevents helical chip formation. By increasing the depth of cut a_p_ to 0.4 mm (Figure 2b,e,h), it is seen that for all values of feed rate, the chip has greater up-curl formation with larger chip curvature radius, and the rings have started to diverge. At a_p_ = 0.65 mm (Figure 2c,f,i), it is seen that for all values of feed rate, chip curling took a helical form with a large pitch to the helix (the distance between two consecutive rings) and larger chip curvature radius. Increasing chip curvature radius due to a deeper depth of cut is because of the greater width of the chip, which increases the resistance to a decreasing chip curve radius. This is due to the bending moment at the free end of the chip acting on the root of the chip in the chip tool contact area, somewhat as a cantilever beam. It may also be related to the elastic recovery of the free chip due to the greater chip cross-sectional area.

With regard to the effect of feed rate on chip shape, increasing the feed rate obviously increased chip curling due to the increase in chip thickness [35], which results in increasing the bending moment in the direction of chip thickness and encourages chip up-curling, as discussed above. Higher feed rates also result in a slight increase in chip curvature, which agrees with the findings reported in [2]. Devotta et al. [2] reported that increasing chip curvature radius is related to the chip–tool contact length, which, according to Iqbal et al. [36], is increased by a higher feed rate.

Figure 4 shows the effect of cutting speed on chip morphology for different depths of cut at a feed rate of 0.2 mm/rev. It was found that a snarled chip was dominant at higher cutting speeds, while chips with up-curl formation and a small radius of curvature were found at a low cutting speed. These results are in agreement with the results reported in [48], which concluded that the chip turned from a helical to snarled ribbon chip with increased cutting velocity and explained this by the reduction in chip thickness with increasing cutting speed, which prevented the formation of chip up-curl. This can also be attributed to low tool–chip interface friction and smaller chip–tool contact length at higher cutting speeds [49], which encourages a straight chip to form and prevents chip up-curl.

Looking at Figure 4e,f,h,i, it is seen that the interaction of cutting speed and depth of cut has a significant effect on chip shape. The combination of higher cutting speeds and larger depths of cut results in snarled chips because of chip side-curl formation. This occurs due to the increasing difference in chip velocity in the direction of chip width as cutting speed and depth of cut increase.

To elaborate on this phenomenon, Figure 5a shows the beginning of a chip side-curl formation with higher chip curve radius at v_c_ = 300 m/min, a_p_ = 0.4 mm and f = 0.1 mm/rev, which results in a snarled chip entangled with the workpiece (Figure 5b). The combination of high cutting speeds and greater depth of cuts showed this unwanted effect. However, a greater depth of cut with the lowest cutting speed showed a different mechanism that resulted in the formation of up-curl chips, as shown in Figure 4d,g. Higher cutting speeds (v_c_ = 200 and 300 m/min) at the lowest depth of cut (a_p_ = 0.15 mm) lead to the formation of up-curling chips, as shown in Figure 4b,c. This can be explained by the diminishing effect of chip side-curl formation at this small depth of cut and these higher cutting speeds. Moreover, it was also noticed that the tendency for chip breaking increased under these conditions (Figure 4b,c) due to greater deformation of high cutting speed on chips with small cross sections. However, at the lowest cutting speed (Figure 4a), the chip was found to be more ductile and continuous, and as a result, it became more difficult to break. At higher cutting speeds, a more brittle chip was produced due to higher shear strain [50]; as a result, there was a tendency for the chip to break (see v_c_ = 200 and 300 m/min with 0.015 mm depth of cut, Figure 4b,c).

Figure 6 shows the effect of feed rates and cutting speeds at 0.15 mm depth of cut. As previously explained, at the lowest depth of cut and higher cutting speeds, no side-curl chip is formed but rather, the chip tends to curl to form a helical chip. Increasing the feed rate increased chip curling for all cutting speeds. Figure 6a–c shows the minor effect of a low feed rate, 0.1 mm/rev, on chip curling at all values of cutting speeds, as explained previously. At a feed rate of 0.1 mm/rev and 300 mm/min cutting speed (Figure 6c), the chip tends to be a ribbon which snarls. This is due to a straight chip being formed by a higher cutting speed and low chip thickness, especially with a low feed rate and with partial disappearance of chip up-curl. By increasing the feed rate to 0.2, and then to 0.3 mm/rev, at cutting speeds of 200 and 300 m/min (see Figure 6e,f,h,i), the chips tend to break.

Figure 7 shows the effect of combining higher values of cutting speed (v_c_ = 200, 300 m/min) with a large feed rate (f = 0.3 mm/rev) at a low depth of cut (0.15 mm) on the improvement of chip breakability and evacuation during machining.

Figure 8 shows chip tangling with the workpiece at higher values of cutting speed and depths of cut for different feed rates. The tangling is due to the significant effect of chip side-curl with a large radius of curvature, which prevents chip up-curl and produces helical chips. Although increasing the feed rate encouraged the formation of chip up-curl at low cutting speeds, and even at a higher cutting speed with a low depth of cut, there was no effect of feed rate on the formation of chip up-curl when the higher cutting speeds were combined with greatest depth of cut. The enhanced chip side-curl formed due to the interaction of a higher cutting speed and large depth of cut overcame the effect of feed rate on chip up-curl.

It was concluded that the interaction of the three process parameters, feed rate, cutting speed and depth of cut, has a significant effect on chip control and breaking. A summary of the results is presented in Figure 9. Safe combinations of cutting speed, depth of cut and feed rate regarding chip evacuation are shown in green and risky combinations in red. It is recommended to avoid combinations of high cutting speed and high depth of cut when machining Ti-6Al-4V. A higher feed rate with a higher cutting speed at a low depth of cut is recommended for good chip breakage.

### 3.2. Comparison between Chip Morphology at Micro and Macro Level

When machining titanium alloys, a serrated type of chip (saw-tooth chip, see Figure 10) was found to be the dominant chip formed [51]. Some researchers have reported the formation of a segmented chip when machining titanium alloys due to adiabatic shear band formation [10,52], which is the formation of localized shear bands when the rate of thermal softening overcomes the rate of strain hardening [53]. Other researchers have claimed that the saw-tooth chip is initiated by periodic crack formation rather than adiabatic shear bands [54]. In fact, there is no clear explanation of the formation of saw-tooth chips due to the complexity of the thermal and fracture analysis [50]. The chip formation mechanism is being studied by a number of researchers by both finite element analysis [55] and experiment [56]. As discussed above, chip segment geometries are affected by machining conditions, in addition to such process parameters, as friction between tool and workpiece [57] and the temperature generated due to cutting [58] need to be considered. Few researchers have found a relationship between chip curling mechanisms and chip deformation in the plastic region.

This section discusses the relationship between chip morphology at the micro and macro level for chips which have a different shape to better understand chip curling mechanisms. Figure 10 shows a SEM image of a typical segmented chip produced by turning Ti-6Al-4V alloy, with a feed rate of 0.2 mm/rev, cutting speed of 75 m/min and 0.65 mm depth of cut. The pitch is the distance between two segments, t_max_ is the maximum chip thickness and t_min_ is the minimum chip thickness.

Figure 11 and Figure 12 present comparisons between SEM images of scanned chips produced in two experiments. The first for conditions f = 0.3 mm/rev, v_c_ = 300 m/min and a_p_ = 0.65 mm, and the second for f = 0.2 mm/rev, v_c_ = 75 m/min and a_p_ = 0.65 mm, so that each had a different chip curl mechanism. These figures show chip morphology at the micro scale from different orientations: chip free surface, Figure 11a and Figure 12a; back surface, Figure 11b and Figure 12b; two side views of chip thickness, Figure 11c,d and Figure 12c,d. Figure 11e and Figure 12e show the chip shapes on the macro scale. Figure 11 is for a side-curling chip, for the stated cutting conditions. Figure 12 is for an up-curling chip produced by the stated cutting conditions.

As shown in Figure 11c,d, it was found that the two side views of chip thickness showed different numbers of segments and chip pitch. The chip thickness image in Figure 11d shows a smaller pitch between segments, so, there are more segments on this side than the other, shown in Figure 11c. This reflects a big difference in chip velocity across the chip in the direction of chip width, and this resulted in a chip side-curl formation, which agrees with Bai et al. [4], who concluded that segmentation pitch decreased with increased cutting speed and thus chip velocity, which confirms the present results.

The chip free and back surfaces shown in Figure 11a and Figure 11b, respectively, show fracture cracks on the right side of chip width. This was attributed to the tension acting on this side of the chip due to the dynamics of chip formation and evacuation which generate different stresses on the two sides of the chip, one in tension and the other in compression. No similar mechanism was found in the case of chip up-curling formation (see Figure 12).

The two side views of chip thickness have neglectable differences in pitch and number of segments, which reflected the observation that no chip side-curl occurred (Figure 12c,d). On other hand, looking at the free surface of the chip (Figure 12a), it can be seen that a greater compression of segments occurred toward one plane, marked by the yellow dashed line, which also shows the peak value of chip thickness (t_max_). This can be compared with the relatively uniform segments of maximum chip thickness observed in the case of a chip side-curl (Figure 11a).

The variation of chip thickness across the chip width (Figure 12a), which reaches its maximum value at the yellow dashed line, reflects the greatest difference between tension and compression, which occurred on the back and free surface of the chip, respectively. Also, in the case of chip up-curl (Figure 12b), the back surface showed chipping, which is explained by the friction that occurred between the tool and chip interfaces in this case. This friction can increase tension in the back surface of the chip, which encourages chip up-curl formation (Figure 12e), while no chipping on the back surface was found in the case of chip side-curl (Figure 11b).

Figure 13d–f shows a micro comparison between chips formed in three different experiments, each of which generated chip up-curling with different macro chip curve radii and pitches of helix (see Figure 13a–c). The cutting conditions were (a) f = 0.3 mm/rev, v_c_ = 75 m/min and a_p_ = 0.15 mm, (b) f = 0.2 mm/rev, v_c_ = 75 m/min and a_p_ = 0.4 mm, and (c) f = 0.2 mm/rev, v_c_ = 75 m/min and a_p_ = 0.65 mm.

From the chip thickness analysis (Figure 13d–f), it was found that increasing the pitch of segmentation resulted in a higher chip radius of curvature, as seen in the images at the macro level (Figure 13a–c). This is in agreement with results presented in [50], which reported that the pitch between two segments has a proportional relationship with tool–chip contact length and correlates with chip curve radius. Examination of the SEM images for the three experiments found that the pitch of the helical chips correlated with the position of the maximum chip thickness of the segments (t_max_) along chip width, marked with the dashed yellow line, as illustrated in the free surface images of the chips (Figure 13g–i). The position of the (t_max_) line could be used to explain the formation of conical chips, especially when the maximum compression of chip segments was located laterally along the side of the chip width, and the closer the yellow line is to the chip side, the higher the pitch of the helix produced (see Figure 13c,i). Figure 13j shows more detail of the conical-shaped chip, which is a magnified image of the chip presented in Figure 13c.

By looking at the free surface in Figure 13g, it was found that t_max_ for the segments occurred approximately in the middle of the chip width and led to smaller helix pitches. Figure 13i shows that the t_max_ of segments was found close to the lateral side of the chip, and a larger pitch for the helix was found to more closely resemble a conical shaped chip, which is dissimilar to those presented in Figure 13g. This observation agrees with Fang [18], who reported that increasing the pitch of the helix results in conical chip formation.

### 3.3. Effect of Chip Control on Measured Surface Roughness

Surface roughness is directly affected by machining parameters [59]. Feed rate and nose radius are the most significant parameters affecting surface roughness, followed by cutting speed and depth of cut [60], while a lower feed rate obviously decreases surface roughness [61]. Figure 14 and Figure 15 show the effect of chip tangling on surface roughness during machining, with respect to process parameters.

Figure 14 shows the effect of feed rate on surface roughness for different cutting speeds, when the depth of cut is 0.4 mm. Different trends were observed for the effect of feed rate on surface roughness for the range of cutting speeds used. A feed rate of 0.1 mm/rev for v_c_ = 75 m/min gave better chip evacuation than cutting speeds of 200 and 300 m/min; in the former case, there was chip curling, but for the latter two cases, tangled chips were produced, increasing the surface roughness. The difference in chip formation is proposed as the explanation for why minimum surface roughness was achieved with the lowest cutting speed at the minimum feed rate. However, it is to be expected that the lowest feed rate would give the minimum surface roughness when other parameters are kept constant.

Again, a feed rate of 0.2 mm/rev for v_c_ = 75 m/min gave better chip evacuation than cutting speeds of 200 and 300 m/min. As before, there was chip curling for the lowest cutting speed but chip entanglement for the higher speeds, which would have increased the surface roughness. However, we see the positive effect of cutting speed becoming more important, and the measured roughness for 75 and 300 m/min is not significantly different.

However, when the feed rate increased to 0.3 mm/rev, the lowest cutting speed produced the roughest surface, despite it not producing tangled chips. This can be attributed to the dominant positive effect of the high cutting speed, where high cutting speed gives a lower surface roughness and balances out the negative effect of chip tangling on the generated surface. As seen in Figure 7, the tangling between the chip and workpiece at a higher cutting speed is less significant at both feed rates shown (0.1 and 0.3 mm/rev).

Figure 15 shows the same negative effect on the workpiece surface roughness of chip tangling. Here, for v_c_ = 200 m/min, the optimum surface roughness is obtained with the minimum depth of cut for all three feed rates. For the 0.4 and 0.65 depths of cut with the given conditions, in every case, chip entanglement occurred. For the minimum depth, chip breakage occurred for feed rates of 0.2 and 0.4 mm/rev, suggesting that chip evacuation significantly affected the final surface quality.

## 4. Conclusions

This investigation of the turning Ti-6Al-4V alloys studied chip control. The effect of process parameters, namely, feed rate, cutting speed and depth of cut on chip curling and evacuation, has been presented. Examination of the chip via a SEM was used to compare chip morphologies at micro and macro levels. Surface roughness was measured to determine the effect of chip evacuation on the machined surface. The main conclusions are as follows.

- The interaction between process parameters has a significant effect on chip evacuation, control and breakage.
- Continuous helical chips were found at low cutting speeds and gave good chip evacuation for all values of depths of cut and feed rates.
- Increasing the feed rate and depth of cut resulted in increased chip up-curling, with a greater chip curve radius and pitch of helix at low cutting speeds.
- A combination of a low feed rate of 0.1 mm/rev and the highest cutting speed of 300 m/min led to ribbon snarled chips.
- A combination of higher cutting speed (200 and 300 m/min) and greater depth of cut (0.4 and 0.65 mm) resulted in the chip-side curl becoming snarled, with greater tangling of the chip with the workpiece during the cutting process.
- Higher feed rates of 0.2 and 0.3 mm/rev at higher cutting speeds, with a small depth of cut of 0.15 mm, gave good surface roughness results, with chip breaking and evacuation.
- Examining the generated chips using SEM allowed different chip curling formations, chip side-curl and chip up-curl, to be detected and examined.
  ◦ It was found that large pitch segmentation resulted in increased chip curve radius. Moreover, the pitch of the helix was found to increase with the formation of a helical conical chip.
  ◦ The tangling of chips with the workpiece was found to have a negative effect on the generated surface roughness.

Finally, for improvement of chip control, it is recommended to select a relatively higher feed rate with a smaller depth of cut when machining at higher cutting speeds to enable proper chip evacuation and eliminate tangling between generated chips and the cutting tool and/or workpiece.

## Figures and Tables

**Table 1:** Chemical composition of Ti-6Al-4V alloy.

Element	Ti	Al	V	Cu	Fe	Sn	Si	W
%	89.4	5.74	4.4	0.0685	0.162	0.014	0.0119	0.165

**Table 2:** Machining conditions for the experiments.

Workpiece Material	Ti-6Al-4V Alloy
Insert material	PCD
Feed rate (f)	0.1, 0.2, 0.3 mm/rev
Cutting speed (v_c_)	75, 200, 300 m/min
Depth of cut (a_p_)	0.15, 0.4, 0.65 mm
Cooling condition	Dry

## Data Availability

All the raw data supporting the conclusion of this paper were pro-vided by the authors.
